# Bovine viral diarrhea virus in China: A comparative genomic and phylogenetic analysis with complete genome sequences

**DOI:** 10.3389/fvets.2022.992678

**Published:** 2022-09-02

**Authors:** Pir Tariq Shah, Amina Nawal Bahoussi, Aftab Ahmad, Muhammad Sikandar, Li Xing

**Affiliations:** ^1^Institutes of Biomedical Sciences, Shanxi University, Taiyuan, China; ^2^School of Life Science, Shanxi University, Taiyuan, China; ^3^The Key Laboratory of Medical Molecular Cell Biology of Shanxi Province, Shanxi University, Taiyuan, China; ^4^Shanxi Provincial Key Laboratory for Prevention and Treatment of Major Infectious Diseases, Taiyuan, China

**Keywords:** phylogenetic analysis, recombination, B cell epitope, E2 glycoprotein, China, bovine vial diarrhea virus (BVDV)

## Abstract

Bovine viral diarrhea virus (BVDV), causing bovine viral diarrhea (BVD) in cattle, is one of the highly contagious and devastating diseases of cattle. Since 1980, BVDV has been identified all-over China in a variety of animal species including cattle, camels, yaks, sheep, water buffalo, goats, Sika deer and pigs. In this study, 31 BVDV complete genomes reported in China (from 2004 to 2020) with other 112 genomes reported around the world were comparatively analyzed. Phylogenetic analysis shows that BVDV genomes reported worldwide clustered in three major clades i.e., BVDV-1, BVDV-2, and BVDV-3. The BVDV-1 is genetically the most diverged genotype and phylogenetically classified into 7 sub-clades in our study based on full-length genomes. The China BVDV genomes fall into all three major clades, e.g., BVDV-1, BVDV-2 and BVDV-3. China BVDV-1 clustered into five sub-clades, e.g., 1, 2, 3, 6 and 7, where sub-clade 7 clustered as a separate sub-clade. Full-length genome recombination analysis reveals that the BVDV-1 reported in China appears to be mainly involved in recombination events. In addition, comparative analysis of E2 proteins between BVDV-1, BVDV-2, and BVDV-3 reveals that the amino acid variations could affect 12 potential linear B cell epitopes, demonstrating a dramatic antigen drift in the E2 protein. These results provide a thorough view of the information about the genetic and antigenic diversity of BVDVs circulating in China and therefore could benefit the development of suitable strategies for disease control.

## Introduction

The bovine viral diarrhea virus (BVDV) causes bovine viral diarrhea (BVD), and is among the most widely spread infectious agents around the world (1–3). The BVD was first observed in 1946 in New York USA (4) and characterized with diarrhea, pyrexia, gastrointestinal erosions, leukopenia, anorexia, depression, and hemorrhages (5). The endemic infections of BVDV have been reported in cattle populations all over the world (6–11), where a variety of other domestic and wildlife animal species got involved as well, including buffalo, sheep, pigs, goats, deer, alpacas, and bison (12–14).

The prevalence of BVDV in cattle herds ranges between 28 and 66% (7, 11, 15), while the persistently infected (PI) cattle plays a bigger role in BVDV maintenance in cattle populations (16). Direct contact between animals is the most typical way of BVDV transmission (17), and the virus is shed in a variety of excretions and body fluids including saliva, tears, milk, nasal discharge, urine, feces, sperm, and uterine flushing (2, 18). The virus transmission can also occur during rectal check-up (19), and artificial insemination or natural breeding of cows with infected bull's semen (20, 21).

BVDV belongs to the *genus Pestivirus in the family Flaviviridae*, having three distinct genotypes e.g., BVDV-1, BVDV-2 (22, 23) and a recently identified BVDV-3 (24, 25). According to the recent viral taxonomy release 2021 by ICTV (International Committee on Taxonomy of Viruses), the BVDV-1 are placed in species *Pestivirus A*, BVDV-2 in *Pestivirus B* and the HoBi-like pestivirus (BVDV-3) in *Pestivirus H* (26). However, the classification of BVDV-1 and BVDV-2 into genetic groups is not acknowledged by ICTV but is frequently employed in researches defining BVDV isolates (8). BVDV was also classified into two biotypes, defined by virus lytic activity in cell culture, known as cytopathic (cp) and non-cytopathic (ncp) (22, 23). The non-cytopathic BVDV strains are the more frequent and always involved in the clinically severe form of the acute infection, whereas the cytopathic BVDV strains are rare and generally associated with mucosal disease outbreaks (23).

BVDV genome is a positive sense, single-stranded RNA of ~12.3 kb in length (27), which contains a single open reading frame (ORF) flanked by untranslated regions (UTRs). The ORF encodes for one large polyprotein that is processed during virus replication by viral and cellular proteases into eight non-structural proteins (Npro, P7, NS2, NS3, NS4A, NS4B, NS5A, and NS5B) and four structural proteins (nucleocapsid protein C, Erns, E1, and E2) (8, 28). The Npro is a viral auto-protease that produces its own C-terminus. In pestiviruses, E2 is the immunologically dominant glycoprotein that is the major inducer of neutralizing antibodies (8, 28). The 5′ UTR is highly conserved and contains an internal ribosomal entry site responsible for viral polyprotein translation (8, 28, 29). The 5′ UTR has mainly been utilized to identify sub-genotypes, along with E2 or Npro-coding regions (8, 25, 30–33). Based on 5′ UTR, 23 sub-genotypes have been reported for BVDV-1, designated alphabetically from 1a to 1u, while 4 sub-genotypes of BVDV-2 have been reported till date around the world (8, 34).

The strategy utilized to control BVD is either systematic or non-systematic, which is a key distinction. The herd-to-herd control is regarded as non-systematic, while actions to lower the overall prevalence are regarded as systematic approach (35). Diagnostic testing and vaccination are all important parts of control and eradication strategies (35). Vaccinating females prior to breeding could be an important technique for minimizing BVD-related reproductive failures and limiting BVDV transmission by reducing the number of new born PI calves (36).

BVDV infection was first described in China during 1980, when Changchun 184 strain was identified from an aborted bovine fetus. Since then, BVDV has spread all-over China, with high prevalence. In China, BVDV has been reported in cattle, camels, yaks, sheep, water buffalo, goats, Sika deer, and pigs (34, 37, 38). According to data from a comprehensive review and meta-analysis, BVDV-1 seroprevalence is high in dairy cattle (57%) (39). According to studies, western China has a high proportion of BVDV-positive cattle, owing to the fact that these areas have historically been the main cattle-producing regions (34, 39, 40). In the last decades, demand for dairy products and beef has dramatically increased in China, which has expedited the development and investment in the cattle industry, as well as increased the cattle transport frequency, resulting in BVDV spread extensively throughout the country (34). Genetic evolutionary studies of BVDV can help guide control strategies, vaccine development, and the identification of plausible infection sites by providing significant information on the diversity of viral strains prevalent in the population. Thus, we reviewed the genetic status of BVDV in China by performing the full-length genome-based comparative analysis of the BVDV strains with BVDV strains isolated around the world.

## Materials and methods

### Dataset

A total of 143 BVDV complete genome sequences reported around the world, including 31 complete genomes reported in China were retrieved from publicly available database GenBank (Supplementary Figures S1, S2). The virus genomes in this study were identified by their GenBank ID, name, country, and year of collection in the database i.e., (GenBank ID: virus name/country-year of collection).

### Construction of phylogenetic tree and genomic similarity analysis

All the 143 BVDV complete genome nucleotide sequences were aligned using MAFFT v.7.309 multiple alignment, implemented in Geneious Prime v.2021.2.2, with default parameters (Algorithm: FFT-NS-2, Scoring matrix: IPAM/K=2, Gap open penalty: 1.53, Offset value: 0.123). The alignments were manually curated, and an unrooted Maximum likelihood (ML) phylogenetic tree was constructed using the software Iqtree v.1.6.12 (41). There was a total of 12,011 positions in the final dataset. The best fit substitution model (GTR+F+R4) suggested by jModel test (42), was used for the construction of tree with the 1,000 bootstraps replicates. All the bootstrap values were indicated at each node of the phylogenetic tree. The tree was visualized and modified using Figtree (www.figtree.org). The similarity map of full-length BVDV genome was generated using the software SimPlot v.3.5.1 (43).

### Recombination analysis

The recombination events between the 143 full-length BVDV genomes were analyzed using an automatic Recombination Detection Program 4 (RDP4) software package (44). The potential recombination events were identified using each of the 7 algorithms, i.e., PhylPro, RDP, SiScan, GENECONV, Chimaera, Bootscan, and MaxChi embedded in the RDP4.

### Three-dimensional structure and linear B cell epitopes prediction of E2 protein

The BVDV E2 protein's Linear B cell epitopes were predicted using BepiPred-2.0 server (45). The BepiPred v2.0 server operates under the immune epitope database (IEDB) and predicts the B cell epitope from a protein sequence, running a Random Forest algorithm skilled on amino acids of epitopes and non-epitopes defined from crystal structures. Residues with scores >0.5 threshold value were predicted to be an epitope part and were colored in yellow on the graph (residue scores were shown on Y-axes and residues positions in the sequence were shown on X-axes). We considered only conserved segments of at least 5 amino acid residues that were predicted by BepiPred-2.0 as possible epitopes.

The Iterative Threading Assembly Refinement (I-TASSER) server was used to predict the tertiary structure of the BVDV E2 protein (46–48). The I-TASER is a hierarchical method for predicting protein structure and structure-based function annotations. We first identified the structural templates from the RCSB protein data bank (PDB), following multiple threading approach LOMETS. The full-length atomic models were constructed using iterative template-based fragment assembly simulations.

## Results

### Phylogenetic analysis of full-length nucleotide sequences of BVDV

**Figure 1:**
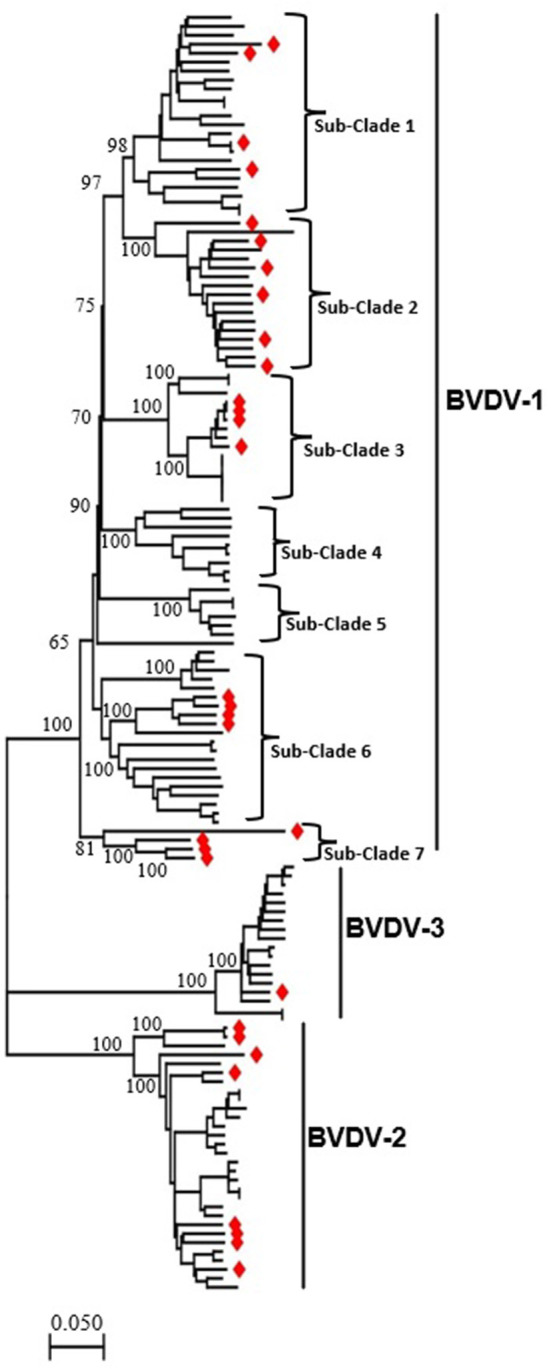
The phylogenetic tree based on the full-length genome sequences of BVDV strains reported around the world, including China. An ML phylogenetic tree of 143 full-length BVDVs genome classifying all the strains into three major clades (BVDV-1, BVDV-2, and BVDV-3). The major clades and sub-clades of the BVDV are indicated. The percentage of replicate trees in which the associated taxa clustered together in the bootstrap test (1,000 replicates) are indicated at each node. The evolutionary distances were computed using the best fit substitution model (GTR+F+R4). Viruses isolated in China are labeled with red diamonds at the tips of the tree. The detailed information of BVDVs in the ML phylogenetic tree is shown in the Supplementary Figures S1, S2.

To determine the genomic characteristics, relationship, and variation of BVDV in China, we retrieved 31 full-length genomic sequences isolated in China during 2004–2020 and 112 isolated around world from the NCBI GenBank database. BVDV-1 strain R3572-90 (GenBank ID: MW655629.1) reported in Switzerland in 1990, was included as the earliest genetic information. The BVDV isolated in China was found in all three major clades representing BVDV-1, BVDV-2 and BVDV-3, respectively, indicating the high genetic diversity of this virus in China (Figure 1, Supplementary Figures S1, S2). The BVDV-1 can further be divided into seven sub-clades. There is no standard nomenclature available for BVDVs classification on sub-genotype level and we labeled all the clusters in the phylogenetic tree obtained in this study as sub-clades from sub-clades 1 to 7 (Figure 1, Supplementary Figures S1, S2). The China BVDV-1 strains nested into five sub-clades, e.g., subclades 1, 2, 3, 6 and 7, that include four, six, four, four and four strains, respectively. Similarly, BVDV-2 included eight, while BVDV-3 included only one strain reported in China (49). The BVDV-1 sub-clade 7 emerged as a new evolutionary branch and consisted of only 4 BVDV strains that were reported in China during 2004–2020, which were genetically more distant from the other sub-clades of BVDV-1. All the other sub-clades contain many BVDV-1 variants of more than one sub-genotype.

### Genetic variation in BVDV isolates in China

**Figure 2:**
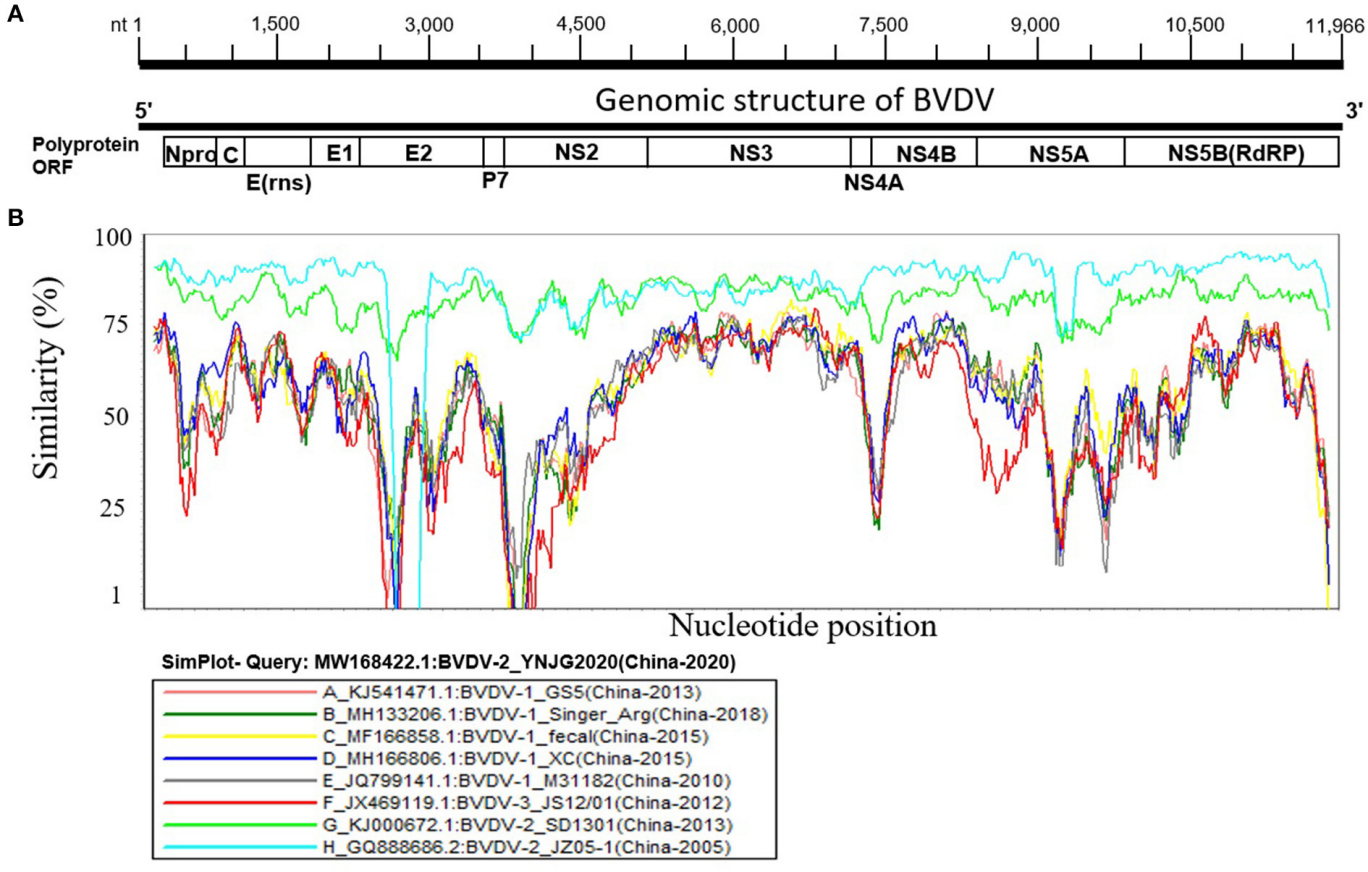
Similarity map of the full-length genome sequence of representative BVDVs isolated in China. **(A)** Schematic diagram of the complete BVDV genome structure. From the 5′ end to the 3′ end is replicase polyprotein ORF encoding proteins Npro, C, Erns, E1, E2, P7, NS2, NS3, NS4A, NS4B, NS5A, and NS5B. **(B)** SimPlot similarity analysis results using BVDV-2 isolate YNJG2020 (GenBank ID: MW168422.1) as the query sequence to compare with the eight other representative strains isolated in China.

BVDV is highly diverged in China, indicating its high genomic sequences variability. The genomic similarity analysis was then performed to further explore the genetic characteristics and relationships using SimPlot v.3.5.1 software. The BVDV genome has one ORF of ~12,218 bp in length, flanked by ~290 bp UTR and encodes for one large polyprotein that is processed by viral and cellular proteases into eight proteins, e.g., Npro, C, Erns, E1, E2, P7, NS2, NS3, NS4A, NS4B, NS5A, and NS5B (Figure 2A). Nine BVDV full-length genomes were included, each representing different lineage of the three major genotypes isolated in China, shown as YNJG2020 (GenBank ID: MW168422.1), (A) GS5 (GenBank ID: KJ541471.1), (B) Singer_Arg (GenBank ID: MH133206.1), (C) Fecal (GenBank ID: MF166858.1), (D) XC (GenBank ID: MH166806.1), (E) M31182 (GenBank ID: JQ799141.1), (F) JS12/01 (GenBank ID: JX469119.1), (G) SD1301 (GenBank ID: KJ000672.1), and (H) JZ05-1 (GenBank ID: GQ888686.2) (Figure 2B). The full-length genome sequence of BVDV-2 isolate YNJG2020 (GenBank ID: MW168422.1) was used as a query strain. In agreement with phylogenetic analysis, BVDV-2 show significantly lower genomic similarity with BVDV-1 and BVDV-3 throughout the genome (Figure 2B). Furthermore, the E2 and NS2-coding genomic regions demonstrated the lowest similarity compared with query strain, indicating their hyper-variability, in particular for BVDV-1. Genome wide similarity analyses showed some regions of the genomic sequences had very higher similarity compared to the whole genome (Figure 2B). The high similarities in the genomic regions of distantly clustered variant in phylogenetic tree indicated recombination events in these variants, which prompted us to carry out genome wide recombination analysis of those BVDV strains.

### Recombination analysis of BVDV isolates reported in China

**Figure 3:**
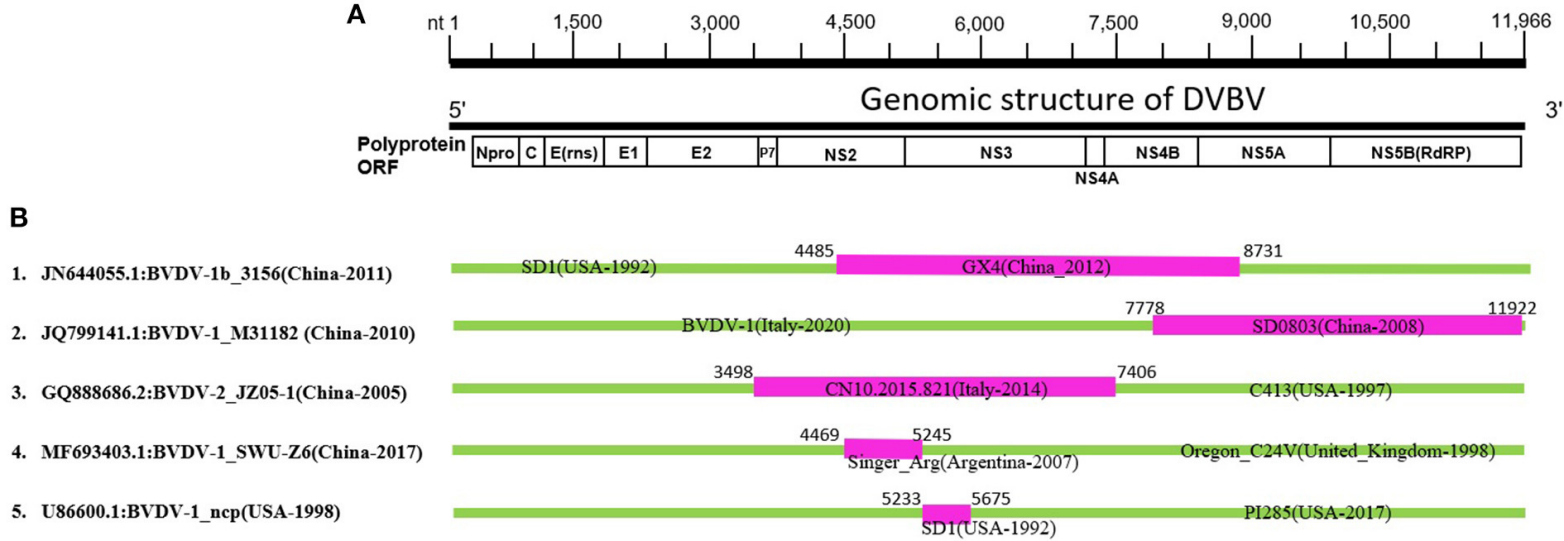
Genetic recombination analysis of 143 full-length BVDV genomes. **(A)** Diagram showing the full-length genome of BVDV and the corresponding regions encoding Npro, C, Erns, E1, E2, P7, NS2, NS3, NS4A, NS4B, NS5A, and NS5B. The numbers indicate the nucleotide positions relative to the genome of BVDV-1 (GenBank ID: JQ799141.1, China-2010). **(B)** Schematic representation of the five potential recombination events listed in Table 1. The recombination event serial number and the description of potential recombinants (GenBank ID: virus name/country-collection year) are show on the left. The filled pink and green blocks represent the DNA regions from minor and major parent viruses, respectively. The numbers on the top of filled green blocks indicate the nucleotide positions of breakpoints relative to the genome sequence of corresponding recombinant viruses on the left.

Recombination in the genomes is one of the major sources of genetic variations and evolution of viral genomes. The phylogenetic tree of BVDV constructed based on full-length nucleotide sequences isolated in China during 2004–2020, and a group of viral isolates emerged to be clustered into a new branch named as sub-clade 7 of BVDV-1 (Figure 1, Supplementary Figures S1, S2). This indicates a rapid divergence and variation in the genomes of BVDVs that could lead to emergence of new variants. We analyzed the recombination events in the full-length viral genomes that were used in phylogenetic analysis (Figure 1, Supplementary Figures S1, S2). Five recombination events were identified for BVDV worldwide (Figure 3, Table 1), in which four recombinants in Events 1–4 were isolated in China (GenBank IDs: JN644055.1, JQ799141.1, GQ888686.2, and MF693403.1, respectively) and one isolated in USA (Event 5, GenBank ID: U86600.1). Four of five recombination events occurred mainly between the sub-clades 1, 2 and 7 of BVDV-1, and one BVDV-2 (Figure 3, Table 1), indicating that the BVDV-1 recombination is driving the viral genetic variability and diversity in China. Furthermore, the beginning and ending breakpoints of recombination were mainly located at the 3,400 bp to the 3′ end of the BVDV genome, the region that encodes for p7, NS2, NS3, NS4A, NS4B, NS5A, and NS5B (RdRP) (Figure 3).

**Table 1:** Identification of 5 potential recombination events in the complete genome of BVDVs isolated during 2004–2020 using RDP4 software package.

**Event serial**	**Recombinant**		**Minor parent**		**Major parent**		**Detection methods**						
**number**	**GenBank ID: Virus name (Country-Year)**	**Clade**	**GenBank ID: Virus name (Country-Year)**	**Clade**	**GenBank ID: Virus name (Country-Year)**	**Clade**	**R**	**G**	**B**	**M**	**C**	**S**	**T**
1	JN644055.1:BVDV-1b_3156 (China-2011)	Sub-clade 2	KJ689448.1:BVDV-1_GX4 (China_2012)	Sub-clade 2	M96751.1:BVDV-1_SD1 (USA-1992)	Sub-clade 1	+	+	+	+	+	+	+
2	JQ799141.1:BVDV-1_M31182 (China-2010)	Sub-clade 7	JN400273.1:BVDV-1_SD0803 (China-2008)	Sub-clade 7	LR760748.1:BVDV-1 (Italy-2020)	Sub-Clade 1	+	+	+	+	+	+	+
3	GQ888686.2:BVDV-2_JZ05-1 (China-2005)	BVDV-2	MG879027.1:BVDV-2_CN10.2015.821 (Italy-2014)	BVDV-2	AF002227.1:BVDV-2_C413 (USA-1997)	BVDV-2	+	–	+	+	+	+	+
4	MF693403.1:BVDV-1_SWU-Z6 (China-2017)	Sub-clade 1	DQ088995.2:BVDV-1_Singer_Arg (Argentina-2007)	Sub-Clade 1	AF091605.1:BVDV-1_Oregon_C24V (United_Kingdom-1998)	Sub-clade 1	+	+	+	+	+	+	+
5	U86600.1:BVDV-1_ncp (USA-1998)	Sub-clade 2	M96751.1:BVDV-1_SD1 (USA-1992)	Sub-clade 1	MN188074.1:BVDV-1_PI285 (USA-2017)	Sub-clade 2	+	+	–	+	+	+	+

The potential recombination events were identified by each of 7 algorithms (RDP, GENECONV, Bootscan, MaxChi, Chimaera, SiScan, and 3Seq) embedded in the RDP4 package.

R, RDP; G, GENECONV; B, BootScan; M, MaxChi; C, Chimaera; S, SiScan; T, 3Seq. +, verified; –, not verified.

*The major or minor parent may be the actual recombinant due to the possibility of misidentification.

**Figure 4:**
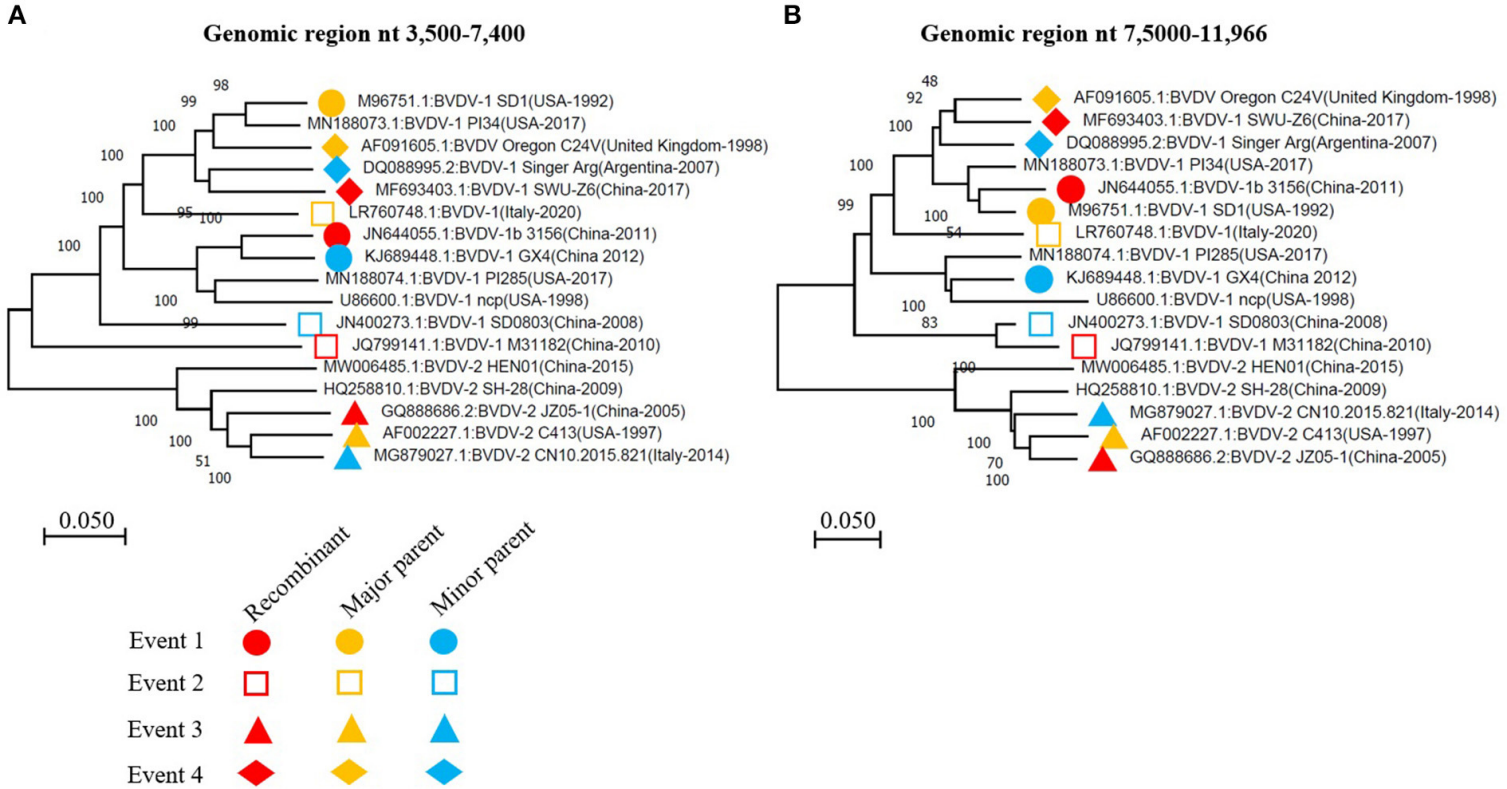
The phylogenetic analysis based on the indicated fragments of BVDV genomes that have been involved in recombination events. **(A)** Phylogenetic tree based on the region nt 3,500-7,400 of BVDV genome. **(B)** Phylogenetic tree based on the region nt 75,000-11,966 of BVDV genome. The viruses involved in recombination events 1, 2, 3, and 4 were labeled with different symbols in the phylogenetic trees constructed using the neighbor-joining method. The recombinant, major parent and minor parent were indicated with red, orange and blue color, respectively. The nucleotide sequence is relative to BVDV-1 (GenBank: JQ799141.1, China-2010).

To further confirm the recombinant events, we constructed two phylogenetic trees of all the BVDV strains involved in recombination, based on two fragments of the genomes. The first fragment from nucleotide 3,500 to 7,400 encodes E2, p7, NS2, NS3 and NS4A, while the second from nucleotide 7,500 to 11,966 encodes for NS4B, NS5A, and NS5B (RdRP) (Figure 3A). The phylogenetic tree based on two fragments or gene regions are not superimposable to each other (Figures 4A,B). For instance, in the first phylogenetic tree (Figure 4A) the recombinant (GenBank ID: JN644055.1) from event 1 nested with its minor parent (GenBank ID: KJ689448), while it nested with its major parent (GenBank ID: M96751.1) in the second tree (Figure 4B). Similarly, the other three Chinese BVDV recombinants also nested with their minor and major parents in the phylogenetic trees based on their recombination (Figures 4A,B). The present results indicate that the BVDVs recombination in different genotypes may lead to the emergence of new sub-genotype variants.

### Variations in linear B cell epitopes of BVDV E2 protein

**Figure 5:**
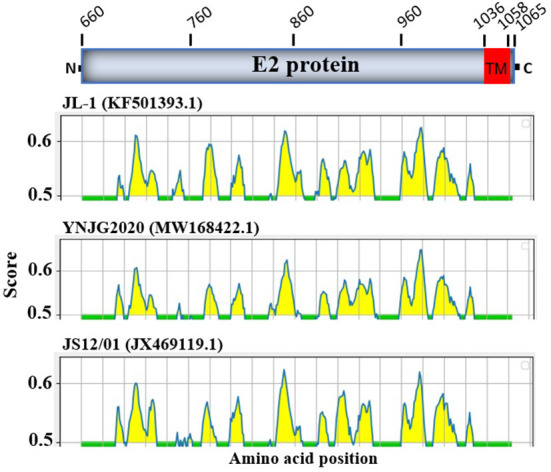
Linear B cell epitope map of the full-length E2 protein of BVDV. Linear B cell epitope distribution in E2 protein were predicted using the BepiPred-2.0 server. The numbers indicate the amino acid position relative to the full-length polyprotein of BVDV 1 strain JL-1 (GenBank: KF501393.1, Protein ID=AII17592.1). E2 protein is aa 660-1065. The X-axes represents the amino acid position, while the *Y*-axes represents the residue score, which is also relative to the diagram on the top. The predicted epitopes are indicated in yellow.

**Figure 6:**
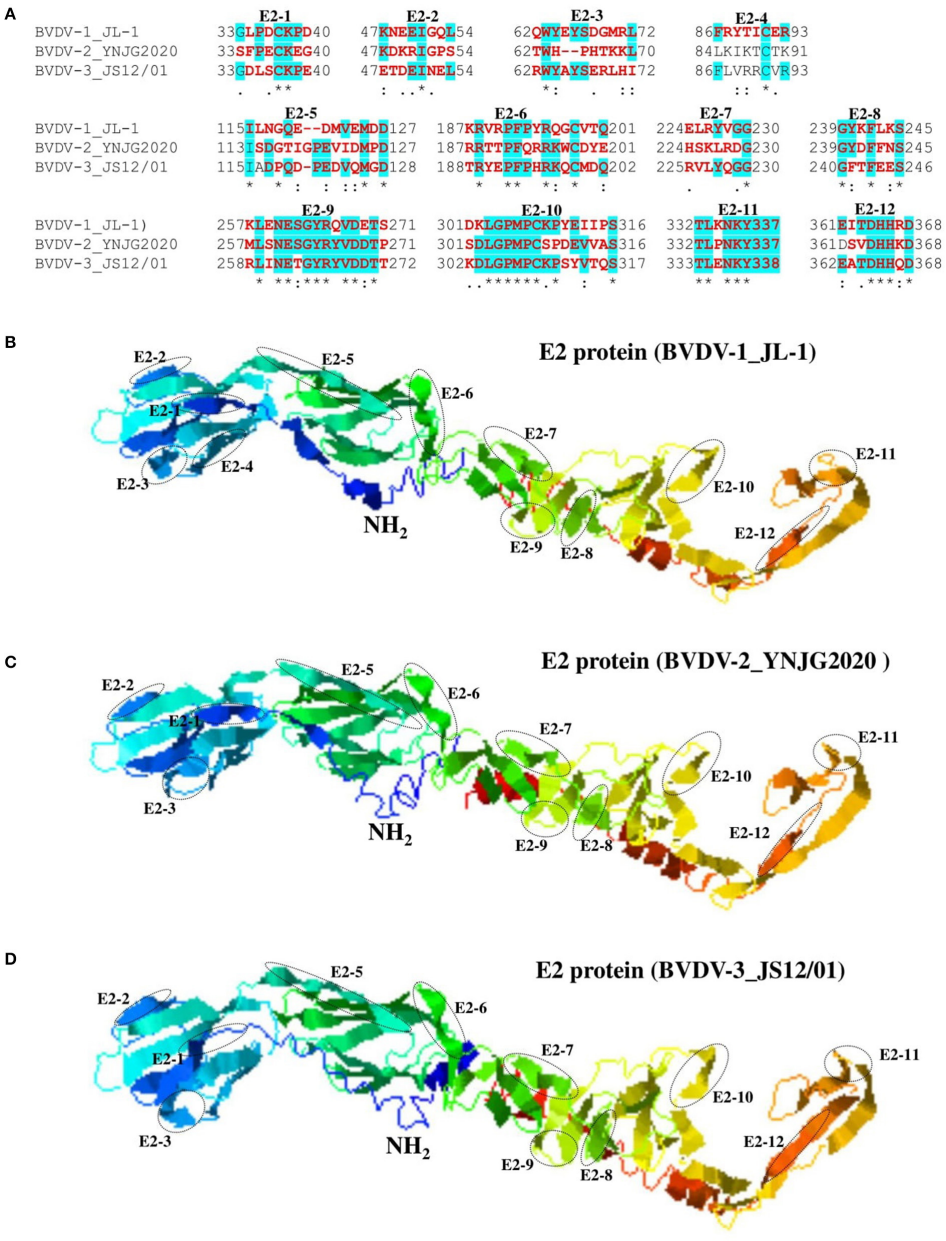
Comparison of linear B cell epitopes affected by amino acid variations in the E2 protein. **(A)** Amino acids of the predicted linear B cell epitope are indicated with bold red letters, while numbers refer to their positions relative to the full-length E2 protein (Protein ID=AII17592.1). **(B)** Three-dimensional view of epitopes in the E2 domain of BVDV-1_JL-1. **(C)** Three-dimensional view of epitopes in the E2 domain of BVDV-2_YNJG2020. **(D)** Three-dimensional view of epitopes in the E2 domain of BVDV-3_JS12/01. The predicted epitopes are circled and indicated.

The E2 protein of BVDV is immunologically dominant and a major target protein for vaccine development (8, 50). The E2 ORF of BVDV was found to be highly variable between BVDV-1, BVDV-2, and BVDV-3 (Figure 2), therefore the effects of detected genomic variations on the antigenicity of E2 protein was assessed. For this purpose, the linear B-cell epitopes on E2 protein of BVDV strains isolated in China were predicted using BepiPred-2.0 epitope prediction server (45). The BVDV isolates reported in China were sorted into three separate evolutionary clades (BVDV-1, BVDV-2, & BVDV-3) based on full-length genome. We analyzed the amino acid sequence and protein structure of E2 protein and found a significant difference in the potential B cell epitopes distribution (Figure 5), especially at position around aa 760, while there were minor differences for the remaining portion of the E2 protein. Therefore, three-dimensional structures of E2 proteins of these viruses were modeled using I-TASSER (46, 47) to visualize the minimal amino acids variations found between evolutionary clades in the potential linear B cell epitopes. The amino acids variations affected 12 linear B cell epitopes of the viruses BVDV-1_JL-1, BVDV-2_YNJG2020, and BVDV-3_JS12/01 (Figure 6). There was a great variation in the amino acid sequences of the potential linear B cell epitopes. The most conserved regions were observed in E2-11 epitope, followed by E2-9 and E2-10 (Figures 6A,B). In summary, the E2 protein may be one of the main factors driving the antigenic drift in the BVDV.

## Discussion

In this study, we conducted the phylogenetic analysis of 143 full-length genome sequences of BVDVs available since 1990 in the NCBI GenBank database, including that reported in China during 2004–2020. The phylogenetic tree showed that all the strains clustered into 3 major clades i.e., BVDV-1, BVDV-2, and BVDV-3 as acknowledge before (22–25). Furthermore, the BVDV-1 can further be divided into 7 sub-clades, with each may contain viruses from different sub-genotypes. Thirty-one BVDVs reported in China have been clustered in three major clades i.e., BVDV-1, BVDV-2 and BVDV-3, indicating the wide genetic diversity of BVDVs in China. Moreover, we found that genomic recombination drives the BVDV-1 genotype expansion and boost the novel evolutionary lineages generation.

In contrast to the previous studies that classified the BVDVs phylogenetically based on single gene (34, 40), we used the full-length genome sequences to construct the phylogenetic tree of the viruses, providing robust support for all the major clades and sub-clades. The present phylogeny does not represent a new classification of BVDV, but provides the first information about the phylogeny of BVDVs based on whole genome sequences. The BVDV-1 was classified into 23 sub-genotype based on single gene or partial sequences in the previous studies (8, 34), while they were clustered into 7 main sub-clades in our study. The present study also describes that each sub-clades contain more than one sub-genotype of BVDV-1. For instance the sub-clade 6 contain viruses belong to sub-genotype BVDV-1m (ID: AF526381.3) (51), sub-clade 7 contain viruses previously reported as sub-genotype BVDV-1u (ID: JQ799141) (37) and BVDV-1q (ID: KC695810) (52).

Most of the BVDV strains isolated in China during 2004–2020 belong to BVDV-1 genotype in our study. The results are consistent with previous studies reporting sub-genotypes of BVDV-1 circulating in China to be more prevalent (8, 37), where three strains (BVDV-1a, c and m) were more dominantly accounting for 80.67% of BVDV-1 reported in China (34). We found that the 31 BVDVs reported in China have been clustered in three major clades i.e., BVDV-1, BVDV-2 and BVDV-3, which is consistent with the previous studies of classification based on partial sequences (40, 49, 53). A new sub-clade emerged in the BVDV-1 (indicated as sub-clade 7) clustering four BVDV-1 strains all belong to China reported during 2004–2020, which are genetically more distant from the other sub-groups of BVDV-1 (Figure 1, Supplementary Figures S1, S2).

Furthermore, we performed recombination analysis for all BVDVs viruses in this study and found that the BVDV genome reported in China is characterized by high level of the recombination. A total of 5 potential recombination events were found in the complete genome among which four events involving BVDV strain reported in China. The recombination occurred predominantly in BVDV-1 genotype, indicating that the BVDV-1 genotype viruses remain the major threat to the cattle industry in China. Genomic recombination are the key factors that contribute to virus diversity and evolution of new strains. The recombination not only drives the BVDV-1 genotype expansion, but also serves as a critical factor to boost the novel evolutionary lineages generation that were classified into BVDV 1 sub-clade 2 and 7 and resulted from the genetic exchange between BVDV-1 sub-clade variants. These new recombinants emergence makes the pandemic field more complicated and heterogeneous in China, posing extra challenges to the effective prevention strategies (34).

BVDV is a heterogeneous, single-stranded RNA virus, so genetic and antigenic changes within serotypes are expected. We analyzed the amino acid sequence variation and structural variations in the epitope of the E2 protein of the representative sequences from the three major clades. The E2 protein is an immunologically dominant and highly variable glycoprotein that is the principal target of neutralizing antibodies, making it a prominent vaccination target (8, 50). We found significant number of substitutions in the epitopes of E2 protein that could lead to differential response of host to these viruses. Substitutions and mutations are expected due to lack of the BVDV RNA polymerase proofreading, and previous studies reported that changes occur in the E1 and E2 envelope glycoproteins genes approximately 1.26 × 10^−3^ nucleotide substitutions per site per year (54). This rate of substitution has significant implications, especially when the vaccines efficacy is dependent on the ability of the antibodies and T cells responses generated by the vaccines to prevent the infection (50). The efficacy of these vaccines can be compromised by the mismatches between vaccine strains and field strains. We characterized E2 protein of BVDVs and indicated that some of the epitopes of E2 protein are highly conserved in the three distant major clades that could be used as potential antigen for vaccine production, and as drug target site. The degree of antigenic variation in currently circulating viruses is unknown, making the broadly protective vaccinations development challenging (31). The structural analysis of E2 protein highlighted highly conserved epitopes in all the strains that can be helpful for vaccine target. The E2 proteins evaluation from circulating BVDV-1a strains by previous studies showed 10% of amino acids variance when compared to viruses similar to the currently used vaccine strains (6). The significance of these amino acids variations is still unknown, which may play a role in viral adaptation and resistance against host immune system and the vaccines.

Though BVDV is widely spread in China and is highly prevalent, there is no control and eradication program available for BVDV in this country. The vaccination in China is not mandatory and currently only the inactivated vaccines are commercially available (34). The BVDV infection's outcome is determined by characteristics of the virus such as virulence and antigenic diversity (genotype, strain or biotype), as well as host parameters like immunological status, host species, pregnancy status, and previous infections with other pathogens (15, 55, 56). Aside from the presence of BVDV in persistently infected cattle, there is evidence that the BVDV may maintain long-term or chronic infections in immune-privileged areas such as in circulating white blood cells, central nervous system, in ovary tissues and testes after an apparent recovery from transient infection (57). The present study provides the basic information related to BVDV in China that could be helpful in designing new strategies of eradicating BVDV including new vaccine productions. Collectively, these findings imply that high recombination and E2 protein variations in BVDV may compromise or even eliminate the antibodies neutralizing activities elicited by BVDV-1 E2 proteins in traditional vaccine strains in China. Hence, BVDV E2 protein conserved epitopes-targeted vaccines could play a vital role and may greatly benefit the China's cattle-farming industry.

In summary, the genetic diversity and phylogenetic classification of BVDVs circulating in China are demonstrated in this study, which represents the prevalence of all three major strains BVDV-1, BVDV-2, and BVDV-3 in China. The presence of several recombination events in BVDV viral isolates from China suggests that they play a role in BVDV diversity and the evolution of new variants. The BVDV-1 genotype was found to be the most genetically diverged and appeared to be primarily involved in recombination events. Furthermore, a comparison of E2 proteins from BVDV-1, BVDV-2, and BVDV-3 reveals that amino acid differences can affect 12 potential linear B cell epitopes, demonstrating a dramatic antigen drift in the E2 protein. These findings provide a comprehensive picture of the genetic and antigenic diversity of BVDVs circulating in China, which could aid in the development of disease-control strategies.

## Data availability statement

The datasets presented in this study can be found in online repositories. The names of the repository/repositories and accession number(s) can be found in the article/Supplementary material.

## Author contributions

AN, PS, and LX conceived the study. AN, PS, and AA performed analysis. MS provided assistance in data analysis. PS and AA wrote the manuscript. PS, AA, and LX revised the manuscript. All authors read and approved the final manuscript.

## Funding

The Programme of Introducing Talents of Discipline to Universities (D21004).

## Conflict of interest

The authors declare that the research was conducted in the absence of any commercial or financial relationships that could be construed as a potential conflict of interest.

## Publisher's note

All claims expressed in this article are solely those of the authors and do not necessarily represent those of their affiliated organizations, or those of the publisher, the editors and the reviewers. Any product that may be evaluated in this article, or claim that may be made by its manufacturer, is not guaranteed or endorsed by the publisher.
