# Supplementary material for: Bovine viral diarrhea virus in China: A comparative genomic and phylogenetic analysis with complete genome sequences
Source: Front Vet Sci. 2022 Sep 2;9:992678. doi: 10.3389/fvets.2022.992678 (PMC9478372; doi:10.3389/fvets.2022.992678)
Supplement: Supplementary file 1 [file Data_Sheet_1.PDF]

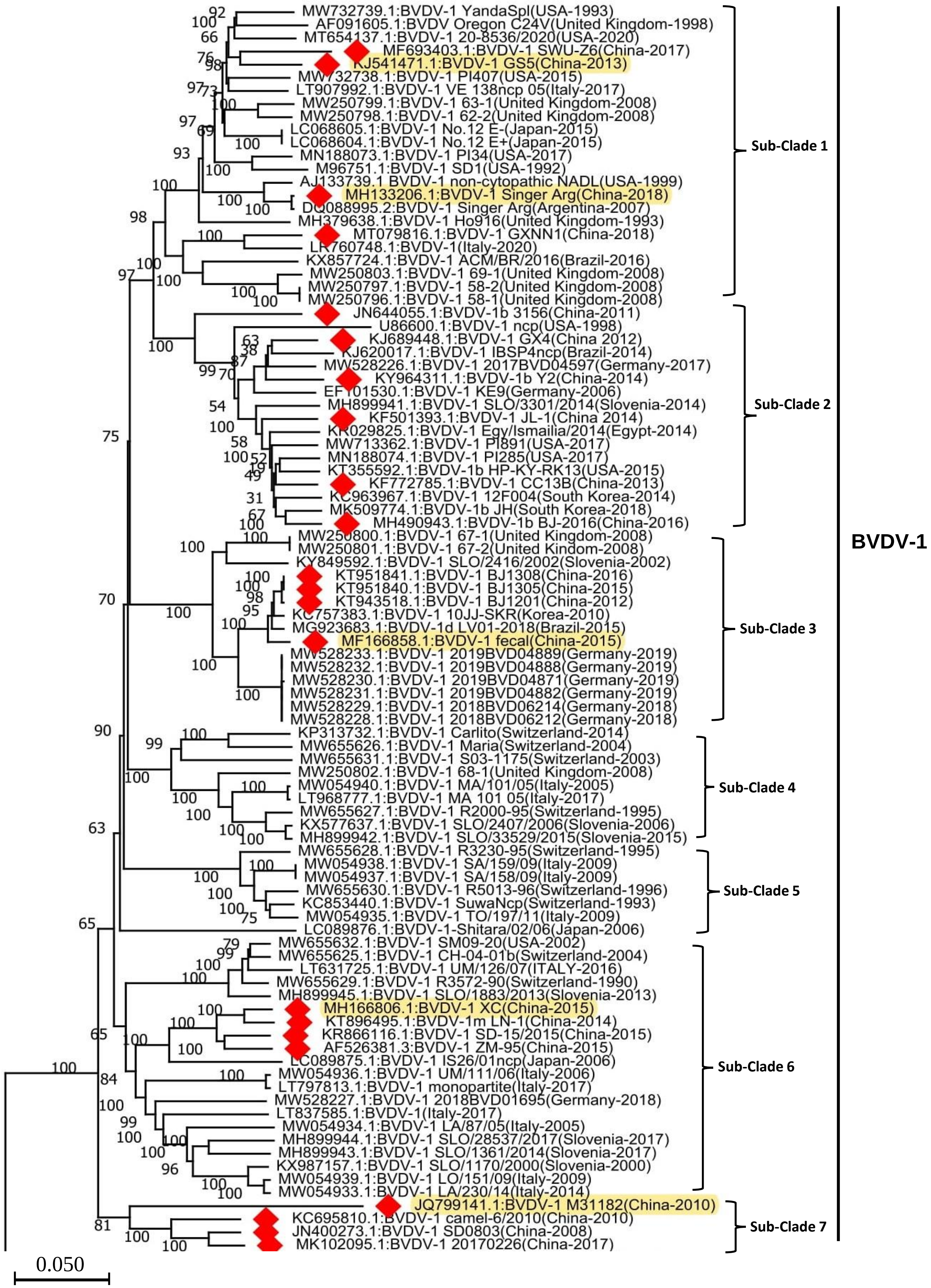

Red diamonds indicate the viruses isolated in China;
Highlighted are viruses selected for Genomic similarity analysis

Supplementary Figure S1

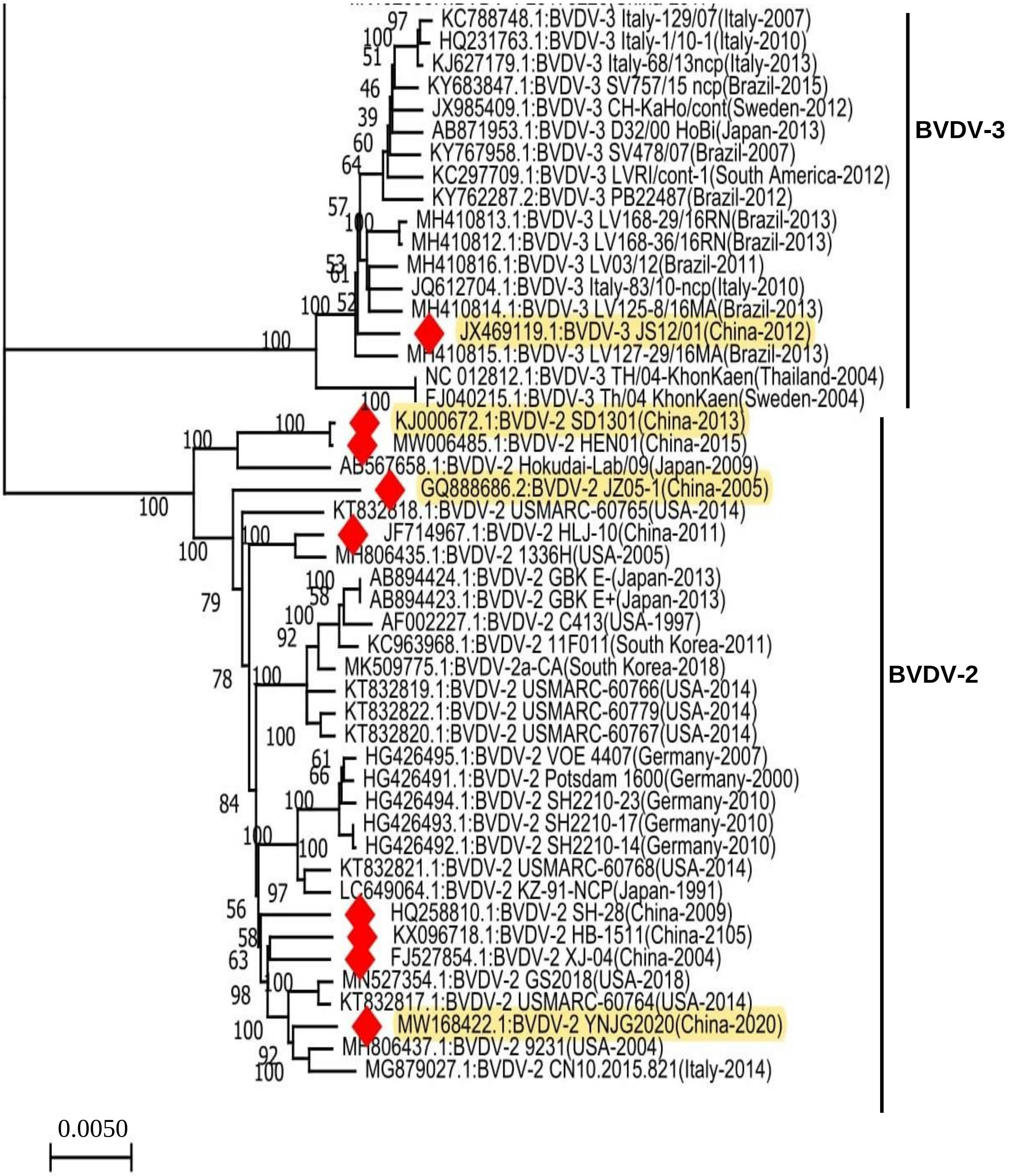

Red diamonds indicate the viruses isolated in China;
Highlighted are viruses selected for Genomic similarity analysis

Supplementary Figure S2
